# Hepatic Hydrothorax in the Absence of Ascites: A Diagnostic Challenge

**DOI:** 10.7759/cureus.16650

**Published:** 2021-07-26

**Authors:** Sangita Kamath, Ashok Sunder

**Affiliations:** 1 Internal Medicine, Tata Main Hospital, Jamshedpur, IND

**Keywords:** hydrothorax, pleural effusion, cirrhosis, liver, ascites

## Abstract

Hepatic hydrothorax is a rare complication of chronic liver disease. It usually occurs in patients with advanced liver disease, portal hypertension, and ascites. On a rare instance, it may be the index presentation of chronic liver disease. Hepatic hydrothorax occurs in approximately 5-6% of patients with cirrhosis. The exact mechanism has not been well defined, but it is frequently thought to be due to the direct passage of ascitic fluid from the peritoneal cavity through the diaphragmatic defects. Treatment involves salt and water restriction and diuretics. Therapeutic thoracocentesis is required in case of respiratory distress. In resistant cases, indwelling pleural catheter (IPC) like PleurX catheter system (Franklin Lakes, NJ: BD) is placed and patients manage their symptoms through intermittent drainage of the pleural fluid. Here we describe an unusual case of hepatic hydrothorax in a patient with rheumatoid arthritis and liver cirrhosis without any ascites, a scenario that has rarely been reported in the literature. The patient underwent thoracentesis thrice but in view of re-accumulating pleural effusion, a pig-tail catheter with underwater seal was inserted. She was then referred to a hepatology center for transjugular intrahepatic portosystemic shunt (TIPS) or liver transplant.

## Introduction

Ascites is a common clinical finding in patients with advanced liver disease with portal hypertension. In about 5-10% of cases, they may develop pleural effusion due to the trans-diaphragmatic movement of ascitic fluid across the pores in the diaphragm. Hepatic hydrothorax is the excessive (> 500 mL) accumulation of transudate in the pleural cavity in such patients with decompensated liver cirrhosis in the absence of cardiopulmonary and renal causes [1]. In rare cases, when the rate of production is less than the absorptive capacity of the peritoneum but more than that of pleura, pleural effusion may develop in the absence of ascites due to negative intrathoracic pressure and diaphragmatic defects. The diagnosis is evident in the presence of known liver disease but might prove more challenging in the absence of history of liver disease. Here we report a rare case of a patient with rheumatoid arthritis and obesity who presented with large transudative pleural effusion in absence of ascites creating a diagnostic dilemma.

## Case presentation

A 50-year-old lady was admitted to our hospital with a history of gradually progressive breathlessness of one-month duration associated with right-sided chest heaviness and mild dry cough. The patient was unable to lie flat without becoming dyspneic. She was a known case of seronegative rheumatoid arthritis (RA) detected two years back and was on oral methotrexate 10 milligram (mg) once per week, tablet sulfasalazine 500 mg twice daily, tablet prednisolone 10 mg once daily, and tablet etoricoxib 90 mg as required. She had no history of fever.

On admission, she was coherent and tachypneic with a respiratory rate of 34 breaths/minute with accessory muscles of respiration working. She had mild pallor but no signs of icterus, clubbing, lymphadenopathy, or pedal edema. There were no other peripheral signs of chronic liver disease. Her body mass index (BMI) was 29.4 kg/m^2^. She was afebrile, her pulse rate was 112/minute, and her blood pressure was 146/82 mmHg. Her respiratory system examination was normal on the left side while on the right revealed reduced chest expansion, stony dull note on percussion, and reduced air entry consistent with right-sided pleural effusion. Examination of the cardiovascular, gastrointestinal, and neurological systems was within normal limits. There were no dilated veins on the abdominal wall. Her initial blood investigations on admission are as shown in Tables 1, 2.

**Table 1:** Laboratory investigations of the patient on the day of admission TLC: total leukocyte count; MCV: mean corpuscular volume; LDH: lactate dehydrogenase; ALT: alanine transaminase; AST: aspartate aminotransferase; ALP: alkaline phosphatase; PT: prothrombin time

Parameter	On admission	Normal range
Hemoglobin (g/dL)	10.2	11.5-16.5
TLC (cells per mm^3^)	4900	4000-11,000
MCV (fL)	93.6	80-100
Platelet count (cells per mm^3^)	98,000	150,000-450,000
Reticulocyte count (%)	1.1	0.5-1.5
Serum LDH (U/L)	533.7	140-280
Total serum protein (g/dL)	6.7	6.6-8.3
Serum albumin (g/dL)	3.07	3.5-5.2
Serum globulin (g/dL)	3.63	2.5-3.5
Serum creatinine (mg/dL)	0.98	0.5-1.5
Total bilirubin (mg/dL)	0.95	0.2-1
Direct bilirubin (mg/dL)	0.7	0.1-0.5
ALT (U/L)	27.1 U/L	5-40
AST (U/L)	94.8 U/L	5-45
ALP (U/L)	140.8 U/L	35-125
PT (seconds)	14	11-15

**Table 2:** Lab investigations (hormonal and antibody assay) TSH: thyroid-stimulating hormone; CLA: chemiluminescent assay; HBsag: hepatitis B surface antigen; HCV: hepatitis C virus

Investigations	Values	Normal range
Antibodies to anti-nuclear antigen (ANA)	Not detected	
Antibodies to double-stranded DNA (dsDNA) - ELISA	Not detected	
Rheumatoid factor (RA)	Negative	
Anti-citrullinated antibodies (anti-CCP)	Negative	
Anti-thyroid peroxidase (TPO) antibodies	1.27 IU/L	< 9 IU/L
Serum TSH (CLA)	3.64 µIU/L	0.3-4.5 µIU/L
HBsag	Negative	
Anti-HCV antibodies	Non-reactive	
Antibodies to HIV 1 and 11	Non-reactive	
Anti-smooth muscle antibody (ASMA)	Negative	

**Figure 1:**
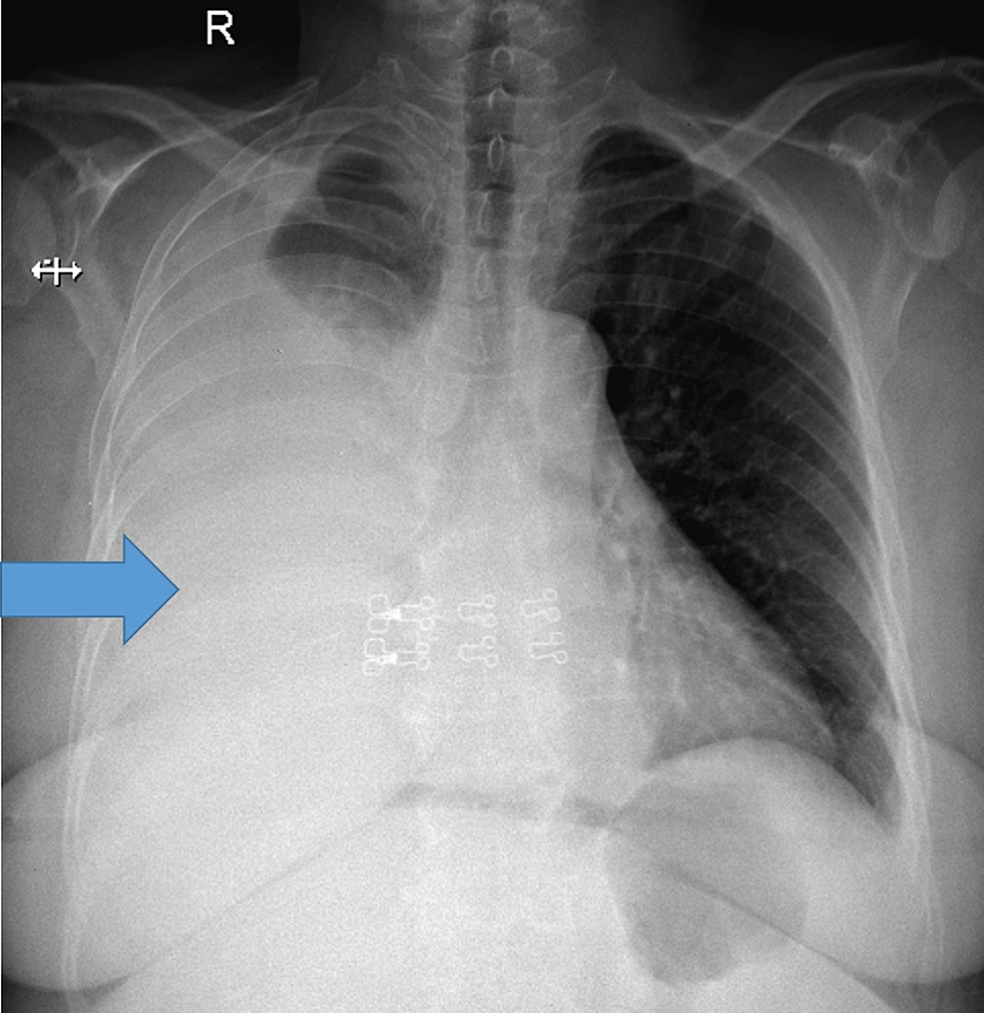
Chest radiograph showing massive right pleural effusion

Her chest radiograph revealed large right pleural effusion (Figure 1).

**Figure 2:**
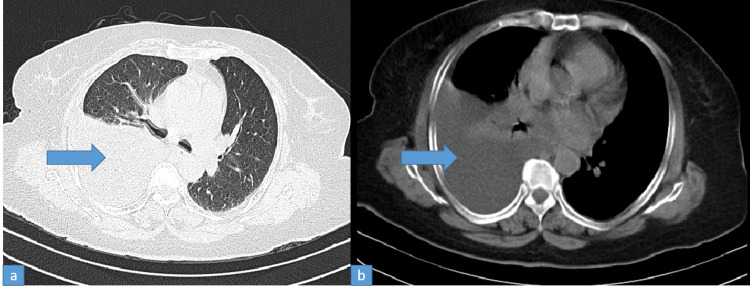
CECT thorax showing right pleural effusion - lung (a) and bone (b) windows CECT: contrast-enhanced computed tomography

Diagnostic tapping of pleural fluid was done. It was pale yellow colored and showed a total of 210 cells with 10% neutrophils, 50% lymphocytes, 30% mesothelial cells, glucose 124 mg/dL (corresponding blood glucose was 130 mg/dL), LDH 71.6 U/L, proteins 0.9 g/dL, albumin 0.4 g/dL, adenosine deaminase (ADA) 2.5 U/L. Smear revealed few neutrophils and mesothelial cells. Malignant cells and parasites were not found. Cultures for bacteria and fungi were negative. Pleural fluid was transudate as per Light’s criteria. Cellblock of pleural fluid did not show any abnormal cells. Gene-Xpert of pleural fluid did not reveal mycobacterium tuberculosis. Ultrasound of her abdomen revealed coarse echotexture of the liver with irregular margins and mild splenomegaly (12.5 cm). There was no ascites. Doppler ultrasound of portal vein and hepatic veins was normal. In view of early cirrhosis of the liver, an upper gastrointestinal endoscopy was done to look for evidence of portal hypertension. It showed portal gastropathy without evidence of varices. Her brain natriuretic peptide (BNP) was 91.2 pg/mL (normal range: 100-400 pg/mL). Echocardiography revealed normal left ventricular ejection fraction, no diastolic dysfunction, normal pulmonary artery pressure, and cardiac valves. Her urine albumin-creatinine ratio (ACR) and microscopy were normal. Contrast-enhanced computerized tomography (CECT) of chest showed moderate right pleural effusion with underlying collapsed lung with few infiltrations in left upper lobe. There were no abnormal lymph nodes or any mass. There was no pleural thickening or nodularity (Figures 2a, 2b).

**Figure 3:**
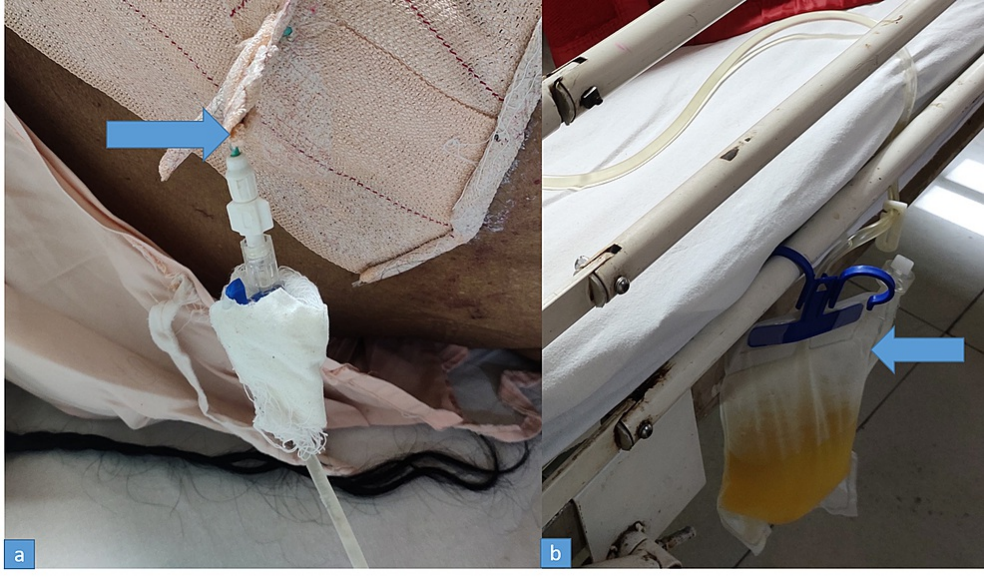
Pigtail catheter (a) connected to underwater seal drainage (b)

A clinical diagnosis of chronic liver disease with model for end-stage liver disease (MELD) score of nine and Child-Pugh class B eight points with hepatic hydrothorax (HH) was made. As she was visibly dyspneic, one liter of pleural fluid was tapped therapeutically. She was also started on intravenous frusemide 40 mg twice daily, oral spironolactone 50 mg twice daily, high protein and low salt (80 mmol/day) diet. However, there was re-accumulation of the fluid and one liter of pleural fluid was tapped again after three days. In the meantime, her diuretics were escalated to frusemide 120 mg/day and spironolactone to 200 mg/day after which she developed hypotension and severe hyponatremia with confusion. Her serum sodium level dropped from 130 mmol/L to 114 mmol/L. Sodium level was corrected gradually with liberal sodium intake and stopping furosemide for three days. Her blood pressure improved but there was re-accumulation of pleural fluid. Chest X-ray showed a large right pleural effusion. As we do not have facility for intercostal pleural catheter (IPC) placement, a pigtail catheter was introduced under radiological guidance (Figure 3a, 3b) and connected to underwater seal.

**Figure 4:**
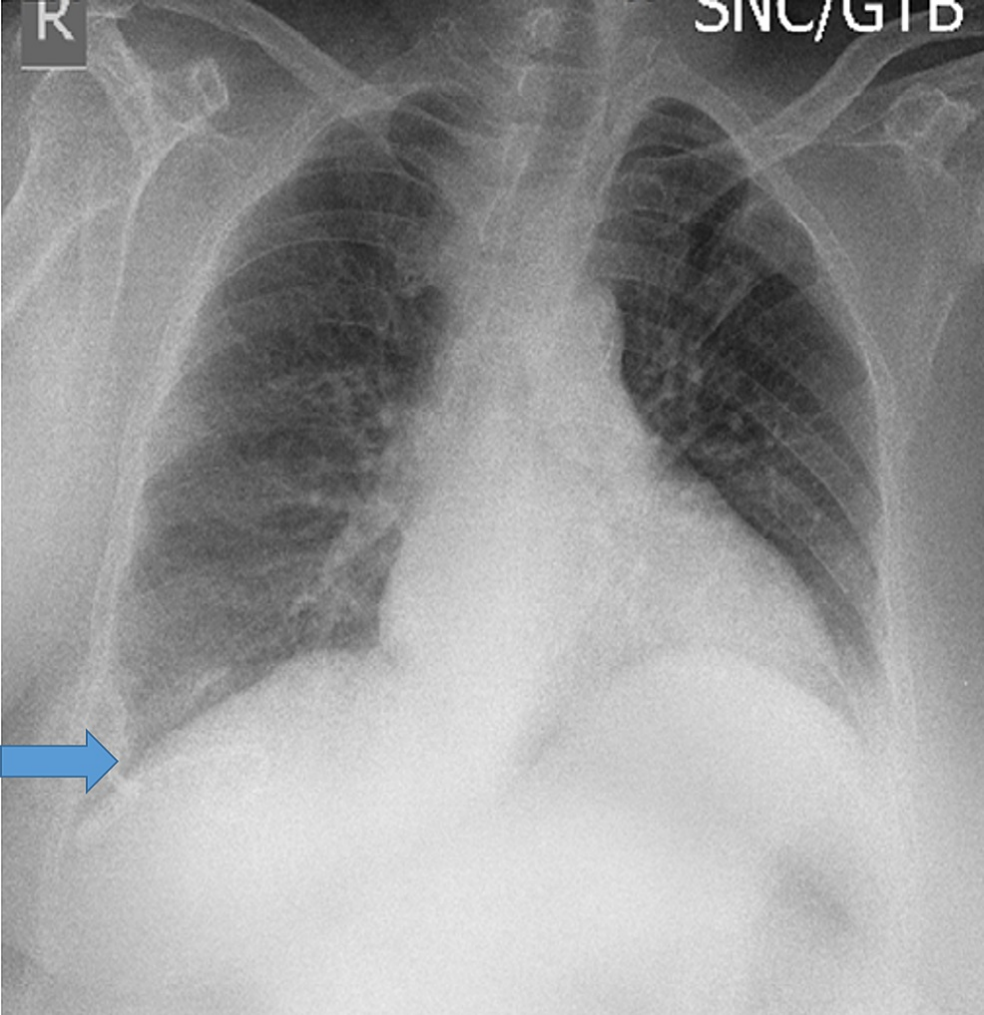
Chest radiograph showing complete lung expansion and drainage of pleural fluid

Two days later, a check chest radiograph showed total drainage of the fluid with complete lung expansion (Figure 4). The patient was then referred to higher hepatology center for further management.

## Discussion

Hepatic hydrothorax (HH) is defined as a pleural effusion, typically more than 500 mL, in patients with liver disease with portal hypertension without coexisting cardiopulmonary disease [1,2]. In our patient, pleural effusion was transudate and there was no evidence of any cardiac, pulmonary, or renal disease, so the liver disease was thought to be the most probable cause of her right-sided pleural effusion (hepatic hydrothorax). The etiology of the liver disease was uncertain. Considering the patient’s BMI, the possibility of non-alcohol-related steato-hepatitis (NASH) seemed likely. Also, she had been on methotrexate for nearly two years for RA, the possibility of methotrexate-induced liver injury leading to acute hepatic decompensation was considered. Differential diagnosis of transudative pleural effusion includes liver failure, renal failure, cardiac dysfunction, urinothorax, and superior vena cava obstruction. Rarely, 5% of malignant pleural effusions may be transudate [2]. The possibility of RA as a cause of her pleural effusion was thought to be unlikely as RA causes exudative pleural effusion with low glucose content.

Hepatic hydrothorax complicates 5-10% of end-stage cirrhotic liver disease [3]. The exact mechanism involved is uncertain. However, it is hypothesized that ascites formed due to portal hypertension and hypoalbuminemia, tracks from the peritoneal space to the pleural cavity through trans-diaphragmatic lymphatics or small defects or fenestrations (usually <1 cm) in the diaphragm [4]. The negative intrathoracic pressure of pleural cavity during inspiration and piston-like effect of the diaphragm favors the unidirectional transfer of fluid across these defects. These defects are difficult to detect, as their size varies from 0.03 mm to 6 mm in diameter. They are more commonly present on the right diaphragmatic cupola as it is developmentally weaker (less muscular) compared to the left cupola [5]. They can be demonstrated by the unidirectional flow of radiolabeled colloid (human albumin labeled with Technetium 99m) through the diaphragmatic channels [6]. Autopsy studies of patients with hepatic hydrothorax also confirm their presence. The majority (85%) of hepatic hydrothorax develop on the right, 13% on the left, and 2% bilaterally [2]. Hepatic hydrothorax can occur without any detectable ascites, due to higher absorptive capacity of the peritoneum as compared to the pleura [7]. Its occurrence in the absence of ascites is rare but has been reported by John et al. [8]. In a study by Badillo et al. among the 77 cases of HH, seven (9%) were without detectable ascites [9]. Although the development of hydrothorax is not related to a particular cause of cirrhosis, alcoholism is commonly seen in many of these patients.

Depending upon the rapidity and the quantity of accumulation of the fluid, the patient may remain asymptomatic or may present in respiratory distress. The size of the effusion is usually moderate but in 6% of the cases, it may be massive. Thoracentesis is recommended in cases of respiratory distress, where urgent drainage of hydrothorax is needed. About 21-26% of patients go on to develop refractory hepatic hydrothorax and may require repeated thoracentesis for symptom control. Medical management includes strict restriction of salt and fluid intake (< 1 L/day) and diuretics like combination of loop diuretics and aldosterone antagonist like spironolactone. The dose of the diuretics is gradually titrated upwards till a maximum of 160 mg/day for frusemide and 400 mg/day for spironolactone is reached. Renal function, electrolytes, and blood pressure should be carefully monitored. However, 20-30% of patients will have persistent effusion despite optimal treatment. In our case, up-titration of diuretics was limited by hypotension and hyponatremia.

Treatment options in resistant cases include placement of chest tube, indwelling pleural catheter (IPC) like PleurX catheter system (Franklin Lakes, NJ: BD), pig-tail catheter, surgical interventions like video-assisted thoracoscopic surgery (VATS), and trans-jugular intrahepatic portosystemic shunt (TIPS) [6]. As we do not have a facility for PleurX catheter placement, we introduced a pig-tail catheter. A recent meta-analysis by Patil et al. related to the use of IPC for non-malignant effusions demonstrated spontaneous pleurodesis rates of 51% [10]. TIPS is the most commonly used second-line treatment for refractory effusion [11]. It relieves portal hypertension by decreasing the portosystemic gradient and can lead to significant improvement in fluid accumulation. In a meta-analysis involving 198 patients of hepatic hydrothorax by Ditah et al., complete response to TIPS was seen in 55.8%, partial in 17.6%, and no response in 21.2% of patients [12]. Liver transplant is the best definitive treatment for refractory HH and improves long-term survival. The purpose of this presentation is to create awareness of this entity among the clinicians. Although an infrequent cause of transudative pleural effusion, HH can cause severe respiratory distress and should be kept in mind as a differential diagnosis in patients even without any history of liver disease or ascites.

## Conclusions

A detailed workup is essential to identify and treat the underlying etiology of transudative pleural effusion. Hepatic hydrothorax is a rare complication in patients with decompensated liver disease and may be the initial presentation of cirrhosis. It can also occur in the absence of ascites, leading to a diagnostic dilemma. Like ascites, management of HH involves strict fluid and sodium restriction and diuretics. However, medical management may not be sufficient to cause complete improvement in all cases and some patients may need thoracentesis or chest tube insertion and early referral to hepatobiliary center for definitive treatment.
